# Doublecortin-Expressing Neurons in Chinese Tree Shrew Forebrain Exhibit Mixed Rodent and Primate-Like Topographic Characteristics

**DOI:** 10.3389/fnana.2021.727883

**Published:** 2021-09-16

**Authors:** Jia-Qi Ai, Rongcan Luo, Tian Tu, Chen Yang, Juan Jiang, Bo Zhang, Rui Bi, Ewen Tu, Yong-Gang Yao, Xiao-Xin Yan

**Affiliations:** ^1^Department of Anatomy and Neurobiology, Central South University Xiangya School of Medicine, Changsha, China; ^2^Key Laboratory of Animal Models and Human Disease Mechanisms of the Chinese Academy of Sciences & Yunan Province, and KIZ/CUHK Joint Laboratory of Bioresources and Molecular Research in Common Diseases, Kunming Institute of Zoology, Chinese Academy of Sciences, Kunming, China; ^3^Department of Neurology, Xiangya Hospital, Central South University, Changsha, China; ^4^Department of Neurology, Brain Hospital of Hunan Province, Changsha, China; ^5^Kunming College of Life Science, University of Chinese Academy of Sciences, Kunming, China; ^6^CSA Center for Excellence in Brain Science and Intelligence Technology, Chinese Academy of Sciences, Shanghai, China

**Keywords:** adult neurogenesis, cerebrum, immature neurons, mammalian evolution, neuroplasticity

## Abstract

Doublecortin (DCX) is transiently expressed in new-born neurons in the subventricular zone (SVZ) and subgranular zone (SGZ) related to adult neurogenesis in the olfactory bulb (OB) and hippocampal formation. DCX immunoreactive (DCX+) immature neurons also occur in the cerebral cortex primarily over layer II and the amygdala around the paralaminar nucleus (PLN) in various mammals, with interspecies differences pointing to phylogenic variation. The tree shrews (*Tupaia belangeri*) are phylogenetically closer to primates than to rodents. Little is known about DCX+ neurons in the brain of this species. In the present study, we characterized DCX immunoreactivity (IR) in the forebrain of Chinese tree shrews aged from 2 months- to 6 years-old (*n* = 18). DCX+ cells were present in the OB, SVZ, SGZ, the piriform cortex over layer II, and the amygdala around the PLN. The numerical densities of DCX+ neurons were reduced in all above neuroanatomical regions with age, particularly dramatic in the DG in the 5–6 years-old animals. Thus, DCX+ neurons are present in the two established neurogenic sites (SVZ and SGZ) in the Chinese tree shrew as seen in other mammals. DCX+ cortical neurons in this animal exhibit a topographic pattern comparable to that in mice and rats, while these immature neurons are also present in the amygdala, concentrating around the PLN as seen in primates and some nonprimate mammals.

## Introduction

Doublecortin (DCX) and other immature neuronal markers are commonly used for the characterization of adult neurogenesis, which predominantly occurs in the subventricular zone (SVZ) and subgranular zone (SGZ) to support cellular replacement in the olfactory bulb via the rostral migratory stream (RMS) and in the hippocampal dentate gyrus (DG) via radial migration (Seki and Arai, 1993, 1995; Nacher et al., 2001; Miller and Sahay, 2019; Abbott and Nigussie, 2020). During this neurogenic process, DCX expression initiates following the proliferation of neuronal stem cells and reduces as the newborn neurons differentiate towards morphologically and functionally mature interneurons in the bulb and the excitatory granule cells in the DG (Brown et al., 2003). Thus, the presence of DCX+ cells in these two sites represents an active state of neurogenesis, which is considered to play a fundamental role in olfaction, learning and memory, and other cognitive and behavioral functions (Anacker et al., 2015; Martinez-Marcos et al., 2016; Grelat et al., 2018; Danzer, 2019; Forest et al., 2019; Miller and Sahay, 2019; Berdugo-Vega et al., 2020).

Outside the SVZ and SGZ, DCX+ cells are found in the brain predominantly over two areas in various mammals including humans, i.e., layer II of the cortex and the paralaminar nucleus (PLN) of the amygdalar complex (Amyg). While it remains an issue of discussion as to whether these cortical and amygdalar DCX+ cells could be produced during postnatal or adult life, they are apparently characteristic of immature neurons, therefore may provide an important cellular substrate for cerebral structural plasticity (for reviews, see Bonfanti and Nacher, 2012; La Rosa et al., 2020b). Notably, layer II DCX+ immature neurons show species differences in regard to their regional distribution, such that they are present prominently in the piriform cortex (Pir) or paleocortex in small rodents, namely mice and rats (Nacher et al., 2001), but occur also in the neocortical areas in many middle and large-brained mammals including guinea pigs (Xiong et al., 2008, 2010; Yang et al., 2015), rabbits (Luzzati et al., 2009), cats (Cai et al., 2009), nonhuman primates (Zhang et al., 2009) and humans (Liu et al., 2008, 2018; Cai et al., 2009; Srikandarajah et al., 2009). Other studies have reported the presence of layer II DCX+ cells in the Pir and neocortex in sheep (Piumatti et al., 2018), members of the Microchiropteran (Chawana et al., 2016) and Megachiropteran (Chawana et al., 2013) species, and Afrotherian mammals (Patzke et al., 2014). In hedgehogs (an insectivore) and some Megachiropteran (i.e., the Wahlberg’s epauletted fruit bat), layer II DCX+ cells are primarily localized to the Pir (Alpár et al., 2010; Bartkowska et al., 2010; Gatome et al., 2010). More recently, a comparative study investigated layer II DCX+ cells in 12 diverse mammals with small-lissencephalic to large-gyrencephalic brains. While virtually absent in rodents, these immature neurons are present over the neocortex in other species, with a linear density increased relative to brain size (La Rosa et al., 2020a).

Similarly, species difference appears to exist among mammals regarding the presence of DCX+ immature neurons in the Amyg. DCX+ amygdalar cells are prominently present in nonhuman primates (Fasemore et al., 2018; Bernier et al., 2020) and humans (Zhang et al., 2009; Martí-Mengual et al., 2013; Liu et al., 2018; Sorrells et al., 2019). They are also found in sheeps (Piumatti et al., 2018), and members of the Microchiropteran (Chawana et al., 2016), Megachiropteran (Chawana et al., 2013), Afrotherian (Patzke et al., 2014; Limacher-Burrell et al., 2018) and Insectivore (Alpár et al., 2010) mammals. However, DCX+ amygdalar cells are not well documented or only infrequently seen in mice, rats, guinea pigs, and rabbits (Nacher et al., 2001; Xiong et al., 2008; Luzzati et al., 2009; Jhaveri et al., 2018).

The tree shrews (*Tupaia belangeri*) including the Chinese tree shrew are a small tropical mammal classified taxonomically as Scandentia, which is phylogenically grouped closely with the Orders of Dermoptera and Primates according to molecular phylogenetics studies (Murphy et al., 2001; Waddell et al., 2001). Phylogenetic analyses based on chromosomal and mitochondrial genomes also indicate that the genetic affinity of Chinese tree shrew is closer to primates than to rodents (Xu et al., 2012; Fan et al., 2013, 2019). Many works have characterized the neuroanatomy and brain development of the Chinese tree shrew (e.g., Wei et al., 2017; Huang et al., 2018; Ni et al., 2018; Huang et al., 2020; Yin et al., 2020). This animal is also increasingly used as experimental models for aging and some brain disorders (Yao, 2017; Fan et al., 2018; Ni et al., 2020; Rodriguez-Callejas et al., 2020; Schäfer et al., 2020). However, to the best of our knowledge, little is known about the presence of DCX+ neurons in the brain of this animal. Therefore, the present study was set to determine the distribution and age-related change of DCX+ neurons in the forebrain of Chinese tree shrew, aiming to extend a basic assessment of adult neurogenesis and immature cortical and amygdalar neurons in this species relative to other mammals.

## Materials and Methods

### Animals and Tissue Preparation

The Chinese tree shrews were used in the present study in compliance with the National Institutes of Health Guide for the care and use of laboratory animals. All experimental procedures were approved by the Ethics Committee of Xiangya School of Medicine, with maximal efforts made to avoid unnecessary use of and stress to the experimental animals.

Male tree shrews aged at 2–3 months (mo)-old (*n* = 2 at 2 mo, *n* = 1 at 2.5 mo and *n* = 1 at 3 mo), 1–2 years (yr)-old (*n* = 1 at 1 yr, *n* = 2 at 1.5 yr, *n* = 2 at 2 yr), and 5–6 yr-old (*n* = 3 at 5 yr and *n* = 2 at 6 yr) were used during the original experiments. Brains from three additional animals aged at 6 mo, 1 yr, and 4 yr, respectively, were examined during the revision of the manuscript. All animals were raised in the tree shrew preservation facility of Kunming Institute of Zoology, Chinese Academy of Sciences, before they were selected for use in this study. The lifespan of tree shrews in captivity is 8–10 yrs. Therefore, the life stages of the animal groups relative to humans could be approximately regarded as early childhood, youth, and mid-age adulthood, according to a 1:8 calculation ratio (Keuker et al., 2004).

Animals were brought from the animal facility into the laboratory on the same day before they were euthanized under overdose anesthesia (sodium pentobarbital, 100 mg/kg, i.p.). Animals were then perfused via the ascending aorta with 0.01 M phosphate-buffered saline (PBS, pH 7.4) for a brief vascular rinse, followed by brain fixation by perfusion with 4% paraformaldehyde in PBS. The whole brain including the OB was dissected out, postfixed in the perfusion fixative overnight, and then cryoprotected in 30% sucrose. In the original experiments, the entire brain was cut frontally in a cryostat at a thickness of 30 μm, with the sections collected serially in PBS into 12 sets of wells in culture plates. The brains from the 6 mo-, 1 yr- and 4 yr-old animals were bisected, with one hemibrain sectioned sagittally and the other horizontally. For double immunofluorescence, six sets of frontal sections at 8 μm thickness were also prepared while cutting brains of the 1–2 yr old animals around the levels of the mid-hippocampus and amygdalar complex. All cryostat sections were preserved in a cryoprotectant at −20°C before immunolabeling (He et al., 2014).

### Immunohistochemistry

Two sets of the sections from each brain were used for DCX immunohistochemistry, while an additional set was stained with Cresyl violet (Nissl stain, data not shown) for histological orientation. The goat polyclonal antibody to DCX (Santa Cruz Biotech, sc-8066, diluted at 1:1,000) was used in the present study, with its specificity well established in previous studies (Nacher et al., 2001; Xiong et al., 2008; Cai et al., 2009; Zhang et al., 2009). DCX immunoreactivity (IR) was visualized with the avidin-biotin-complex (ABC) method. Sections were first treated free-floatingly with 3% H_2_O_2_ for 30 min at room temperature, then with 10% normal horse serum (NHS) in PBS containing 0.1% Triton X-100 for 1 h. Subsequently, sections were incubated with the anti-DCX antibody overnight in PBS with 5% NHS, reacted with biotinylated horse anti-goat secondary antibody at 1:400 for 2 h, and then with the ABC reagents (1:400) for another hr (Vector Laboratories, Burlingame, CA, USA), with immunoreactive product visualized in PBS containing 0.05% DAB (w/v) and 0.003% H_2_O_2_(v/v). Three rinses with PBS were used between the incubations. One set of the DCX immunolabeled sections were counterstained with hematoxylin, which was also used for quantitative analysis. All stained sections were allowed to airdry, dehydrated through ascending ethanol solutions, cleared with xylene, and coverslippered with a mounting medium.

### Double Immunofluorescence

Double immunofluorescence was carried out using the 8 μm-thick cryosections to assess the extent of DCX colocalization with the neuron-specific nuclear antigen (NeuN) and Ki67 in the piriform cortex and amygdala. The sections were preincubated in PBS containing 5% donkey serum and 0.3% Triton X-100 for 1 h to lower nonspecific labeling. Then, they were incubated in the above PBS buffer containing the goat anti-DCX (1:1,000) along with mouse anti-NeuN (Merck-Millipore, MAB377, 1:2,000) or with rabbit anti-Ki67 (Vector Lab., Burlingame, CA, USA; #014-1107, diluted at 1:2,000) overnight at 4°C. On the next day, the sections were washed a few times with PBS and reacted for 2 h at room temperature with Alexa Fluor® 488- and Alexa Fluor® 594-conjugated secondary antibodies (Jackson ImmunoResearch Laboratories Inc., West Grove, PA, USA; 1:200). Finally, the sections were counterstained with bisbenzimide (i.e., Hoechst 33342, Sigma-Aldrich, St. Louis, MO, USA; B2883, 1:50,000) and coverslipped with an antifading medium.

### Imaging and Micrograph Preparation

Sections were initially examined on an Olympus BX51 microscope (CellSens Standard, Olympus Corporation, Japan) for the verification of staining quality. Selected sections were then scanned using the 20× objective on a Motic-Olympus microscope equipped with an automated stage and imaging system (Motic China Group Co. Ltd., Wuhan, Hubei, China), which could yield a final montaged and magnification-adjustable image covering the entire area of a glass slide. The scanned images were further examined using the Motic DS Assistant Lite software from low to high magnifications. The presence and distribution of DCX+ cells in the forebrain at different rostrocaudal, parasagittal and horizontal levels were assessed collectively, with particular attention paid to the IR in the OB, SVZ, RMS, DG, Amyg and the superficial layers of the cerebral cortex. For figure preparation, low magnification images (2×) from the above regions were exported and montaged to construct the global section views at representative neuroanatomical planes, with high magnification micrographs (20×) also obtained from subregions of the same section to illustrate the details of the cellular labeling.

### Quantification, Data Processing, Statistical Analysis, and Figure Preparation

Taking advantage of the grid addition option of the Motic viewer software, we carried out cell count of DCX+ neurons in individual brain regions using a randomized sampling method, which will be illustrated with working examples in the result section. Briefly, the image of a DCX immunolabeled section with hematoxylin counterstain was first viewed relative to neuroanatomical position at a low magnification, such that the entire region (i.e., OB, DG, Pir, or Amyg) for quantification was visible. The numbers (displayed in μm unit) of the most left and most top intersections at the X and Y axes of the grid guide over the region for quantification were identified, and then input into the beginning cells (i.e., A1, B1) in an Excel spreadsheet. The first column (A1, A2, A3…) was then extended down by a stepwise increase of 200 μm until the number in the last cell equivalent to the scale seen on the Y-axis that was at the level of the lowest edge of the quantification area in the section image. The first row (e.g., B1, C1, D1…) was also extended by a stepwise increase of 200 μm until the number in the last cell matched to the scale on the X-axis that intersected with the right edge of the quantification area. An Excel table representing individual 200 × 200 μm^2^ areal divisions of the entire quantification region was thus created. Then, a subset of cells within the Excel table was extracted (saved separately) using either the random “column (one dimensional) or grid (two dimensional) selection” plug-in application, which generated the points on the Y-axis (or X-axis if the image is rotated) or the coordinates on both the X and Y axes, allowing the identification of corresponding sampling zones in the Motic image. For the quantification of DCX+ neurons in the piriform cortex, a subset (10 to 20 according to the rostrocaudal levels, or the size, of the section analyzed) of cells from the first column in the Excel file was randomly determined, indicating the positions along the Y-axis to tag individual sampling zones over layer II in the Motic image. For quantification of DCX+ neurons in the DG, a subset of cells (also 10 to 20 according to the rostrocaudal levels of the section analyzed) in the first two columns of Excel table (i.e., A1, B1) were generated, which provided the positions along the Y-axis to tag the sampling zones along the SGZ in the two edges of the DG in the Motic image. For quantification of DCX+ neurons in the OB and Amyg, a subset of cells (20 to 40 according to the section levels) within the entire Excel table was randomly selected in reference to both the X and Y axes in the Motic images, providing the X and Y coordinates for tagging sampling zones across these two regions. Subsequently, the images were enlarged to a point such that grids in sizes of 200 × 200 μm^2^ or 100 × 100 μm^2^ appeared. Sampling zones in 200 × 200 μm^2^ size were drawn along layer II using the square-drawing tool according to the randomly selected positions at the Y-axis. Similarly, sampling zones in 100 × 100 μm^2^ size were drawn at locations of the SGZ in the two edges of the DG in reference to the randomly selected positions at the Y-axis. For both the OB and Amyg, sampling zones were also 100 × 100 μm^2^ in size, which were tagged according to the randomly determined X and Y coordinates. The above random selection and tagging of the sampling zones were carried out by the experimenters with trained neuroanatomical knowledge. The images were then saved after removing the grids, and the DCX+ neurons in each tagged zone of the anatomical region/structure being analyzed were counted (on a computer screen) at 20× magnification by an experimenter who was blinded to the animal information. Cell count was carried out in four equally distant sections for each brain region, followed by calculation of the mean cell density for each region and each brain, expressed as the number of DCX+ neurons per square millimeter (mm^2^). The cell density data were summarized, with the means and standard derivations (S.D.) calculated for the age groups, while the means were also calculated following normalization of the cell density data to the mean of the youngest group. Finally, the data were imported into Prism spreadsheets (Prism GraphPad 5.01, San Diego, CA, USA), analyzed statistically using one-way ANOVA with Bonferroni’s pair-wise multiple comparisons, and graphed accordingly. *P* < 0.05 was set as the level for a significant difference. Figures were assembled using Photoshop 7.1.

## Results

### Distribution of DCX IR in the Brains of 2–3 Months-Old Animals

**Figure 1:**
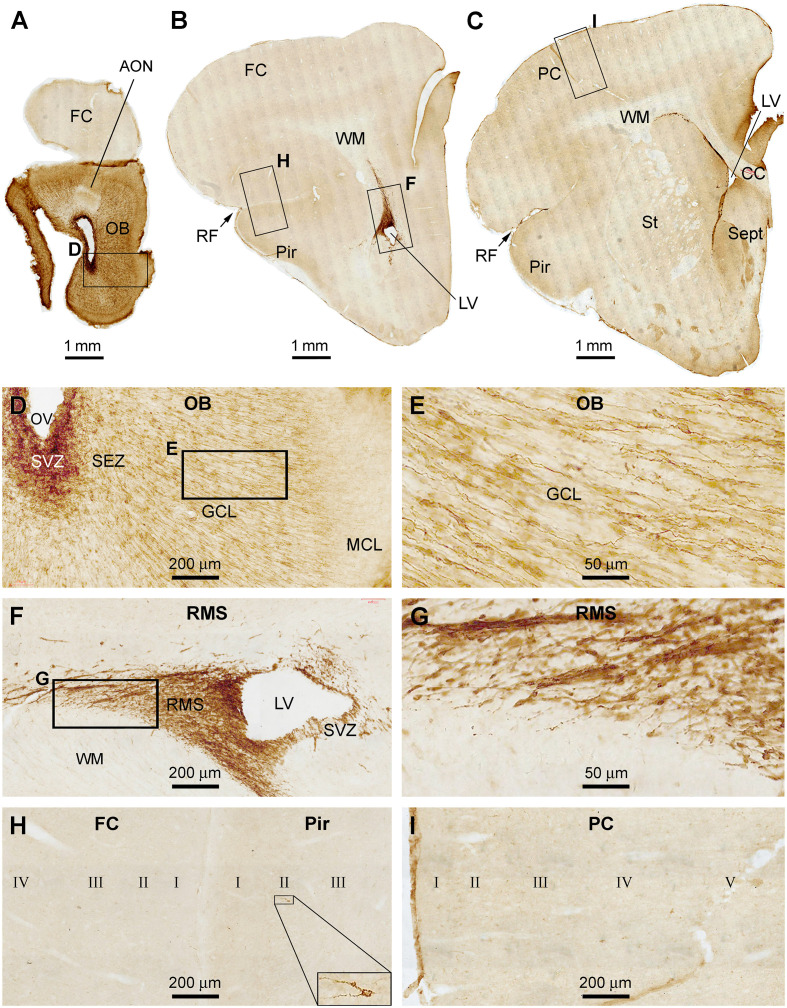
Doublecortin immunoreactive (DCX+) profiles in the rostral part of the forebrain in a 2 months-old Chinese tree shrew. Panels **(A–C)** are low magnification images showing the labeling in the olfactory bulb (OB) at the levels of the anterior olfactory nucleus (AON) **(A)**, the beginning of piriform cortex (Pir) **(B)**, and septum (Sept) **(C)**, respectively. Framed areas are enlarged and illustrated as **(D–I)**. In the OB, DCX+ cells are densely packed at the subventricular zone (SVZ) surrounding the olfactory ventricle (OV). Moderately stained cells are present over the subependymal zone (SEZ) and granule cell layer (GCL) **(D)**. DCX+ granule cells are mostly bipolar or fusiform with their somata and processes oriented centroperipherally **(E)**. Panels **(F,G)** are higher magnification images illustrating DCX+ cellular chains forming the rostral migratory stream (RMS) from the SVZ in the frontal lobe. Panel **(H)** shows part of the frontal cortex (FC) and Pir surrounding rhinal fissure (RF), with no labeled cells in the former, whereas a few labeled cells in the latter (insert). Panel **(I)** shows the lack of DCX+ cells in the parental neocortex (PC). Additional abbreviations: CC, corpus collosum; St, striatum; WM, white matter; MCL, mitral cell layer; I, II…VI: cortical layers. Scale bars are as indicated in individual image panels.

In the anterior part of the forebrain, DCX+ cellular profiles were distinctly present in the OB and the RMS in association with the SVZ in the frontal cortex (Figures 1A–C). Heavily labeled DCX+ cellular profiles occurred across all layers in the bulb, with labeled somata and processes seen in the SVZ/SEZ and GCL. The labeled somata in the GCL were bipolar in shape, oriented radially, and appeared to migrate centroperipherally (Figures 1D,E). It should be noted that the lateral ventricle (LV) extended into the bulb as the olfactory ventricle (OV) in all the three age groups of tree shrews examined in the present study. Thus, the OV appeared as a cavity in sections across the rostrocaudal range of the bulb and connected caudally into the LV in the frontal lobe (Figures 1A–D). Strong and distinct DCX labeling at the SVZ could be continuously traced from the frontal lobe into the center of the bulb (Figures 1A–C). In addition, a wedge-shaped RMS was clearly displayed in the sections passing the caudal part of the bulb to the beginning part of the Pir (Figures 1B,F,G). At high magnification, there were a few strongly labeled neuronal profiles in the Pir, located in layer II. In contrast, no such labeled neurons were found in the frontal or parietal neocortical areas (Figures 1B,C,H,I). Overall, there were no significant immunoreactive profiles seen in the subcortical structures at these frontal levels, including the striatum, septum, and anterior thalamus (Figure 1C).

**Figure 2:**
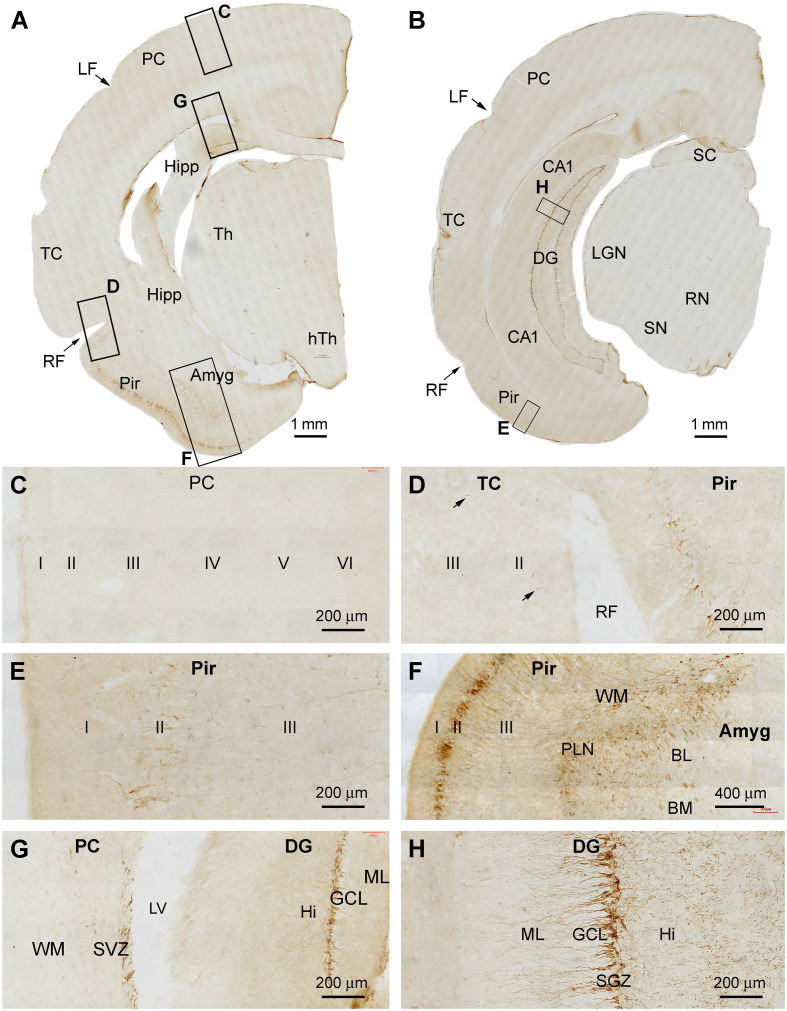
DCX+ cellular profiles in the middle part of the forebrain in a 2 months-old Chinese tree shrew. Panels **(A,B)** are low magnification views of immunolabeled hemispherical sections at the levels passing the rostral end of the hippocampus (Hipp) and the lateral geniculate nucleus (LGN), respectively, with framed areas enlarged as panels **(C–H)**. No labeled cells are seen in the parietal neocortex (PC) and the majority of the temporal neocortex (TC) **(C,D)**, while a few cells could be detected in the temporal cortex surrounding the rhinal fissure (RF) (**D**, pointed by arrows). In contrast, a large number of labeled cells occurs in the piriform cortex (Pir) over layer II **(D–F)**. Abundant labeling is present in the amygdala (Amyg) near its border to the white matter of the adjoining piriform and temporal cortices **(F)**. DCX+ neurons and processes are distinctly present at the subventricular zone (SVZ) next to the lateral ventricle (LV) and in the subgranular zone of the dentate gyrus (DG) **(G,H)**. Additional abbreviations: Th, thalamus; LF, lateral fissure; CA1, Ammons’ horn CA1 sector; SC, superior colliculus; RN, red nucleus; SN, substantia nigra; WM, white matter; PLN, paralaminar nucleus; ML, molecular layer; GCL, granule cell layer; Hi, hilus. I, II…VI: cortical layers. Scale bars are as indicated.

In cerebral sections at the thalamic levels extending from the rostral to temporal ranges of the hippocampal formation, DCX+ cellular profiles were distinctly observed in the DG, Pir, and Amyg (Figures 2A,B). By examining across the cerebral cortex at high magnifications, there were essentially no DCX+ cells found in layer II and other layers over the parietal lobe and the large parts of the temporal lobe (Figures 2C,D), although a few DCX+ cells could be identified in layer II of the temporal cortex around the rhinal fissure (RF; Figure 2D). In contrast, large amounts of DCX+ cells occurred in the Pir, which were packed along layer II from the RF to the basomedial end of this limbic cortex (Figures 2E,F). The dendritic processes of these DCX+ neurons extended into layer I and over layer III towards the white matter (Figure 2F). Deep to the Pir, DCX IR occurred in the Amyg (Figures 2A,F). Most labeled cells were distributed near the amygdalar border to the cortical white matter, therefore consisted of the location of the PLN. The number of the labeled cells reduced as moving to the basomedial (BM) and basolateral (BL) nuclei of the amygdala, while labeled fiber bundles were present in the latter subdivisions (Figure 2F). DCX+ cellular profiles likely reflecting postnatal neurogenesis were clearly present in the DG across the entire hippocampal formation (Figures 2G,H). Specifically, DCX+ cells were densely packed along the SGZ, with their dendritic processes extending into the molecular layer (ML). Fine processes representing axonal profiles were present in the hilus (Figure 2H). No impressive immunolabeled profiles were detected in the subcortical regions, including the diencephalic subareas and the midbrain structures (Figures 2A,B).

**Figure 3:**
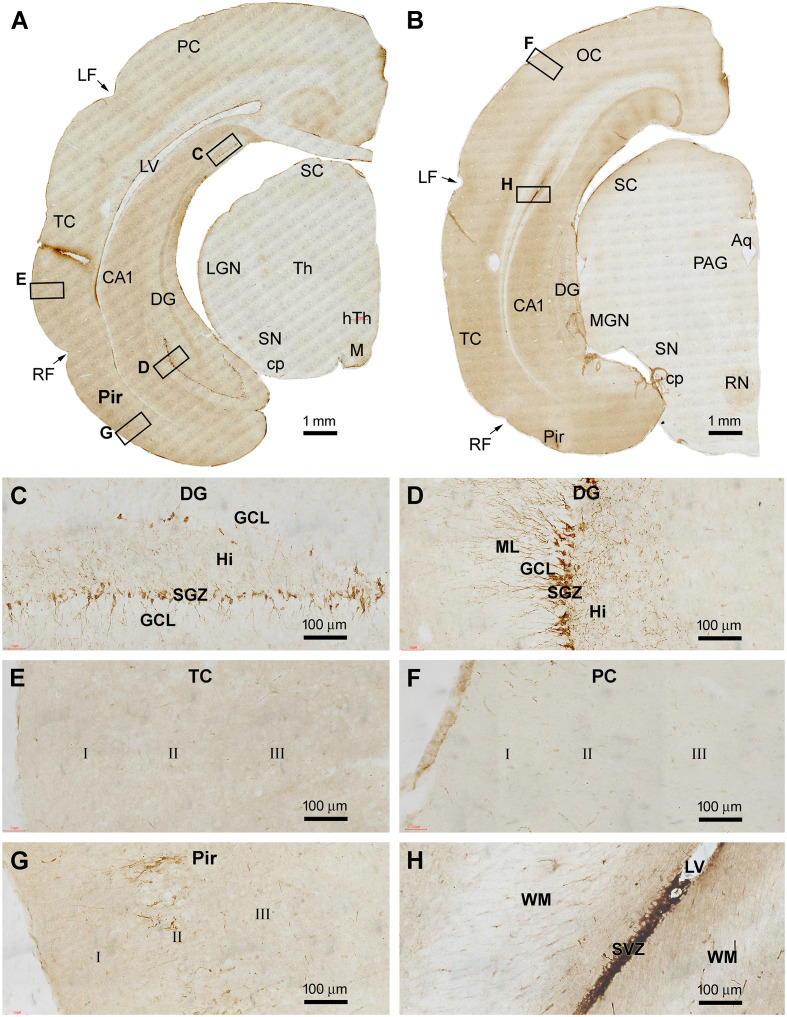
DCX+ cellular profiles in the caudal part of the forebrain in a 2 months-old Chinese tree shrew. Panels **(A,B)** are low magnification views of immunolabeled hemispherical sections at two caudal levels of the hippocampus, with framed areas enlarged as panels **(C–H)**. DCX+ neurons in the dentate gyrus are separately located at the most dorsal and ventral parts of the hippocampal formation **(C,D)**. Again, DCX+ neuronal somata and processes are not found in the temporal and parietal cortical areas **(E,F)**, but are present in the piriform cortex over layer II **(G)**. DCX+ cells are also found at the subventricular zone in the temporal and occipital lobes **(H)**. Abbreviations are as defined in Figures 1, 2, and the following: cp, cerebral peduncle; hTh, hypothalamus; M, mammillary body; OC, occipital neocortex; MGN, Medial geniculate nucleus; RN, red nucleus; Aq, aqueduct; PAG, periaqueductal gray. Scale bars are as indicated.

In cerebral sections at the more caudal levels of the hippocampal formation, DCX IR occurred separately in the dorsal and ventral parts of DG (Figures 3A,B). At high magnification, the immunolabeled cells were densely packed at the SGZ as seen at more rostral levels, with well-stained dendritic processes extending across the GCL into the ML, and fine axonal plexuses in the hilus (Figures 3C,D). Again, there was no DCX IR in the parietal and temporal neocortical regions at these section levels (Figures 3E,F). In contrast, DCX IR was clearly present in the Pir over layer II, with distinctly labeled neuronal somata and dendritic processes seen at higher magnifications (Figure 3G). DCX+ neurons were also present at the SVZ in the temporal lobe (Figure 3H), while little labeling was seen in the midbrain (Figures 3A,B).

### Distribution of DCX IR in the Brains of 1–2 Years-Old Animals

**Figure 4:**
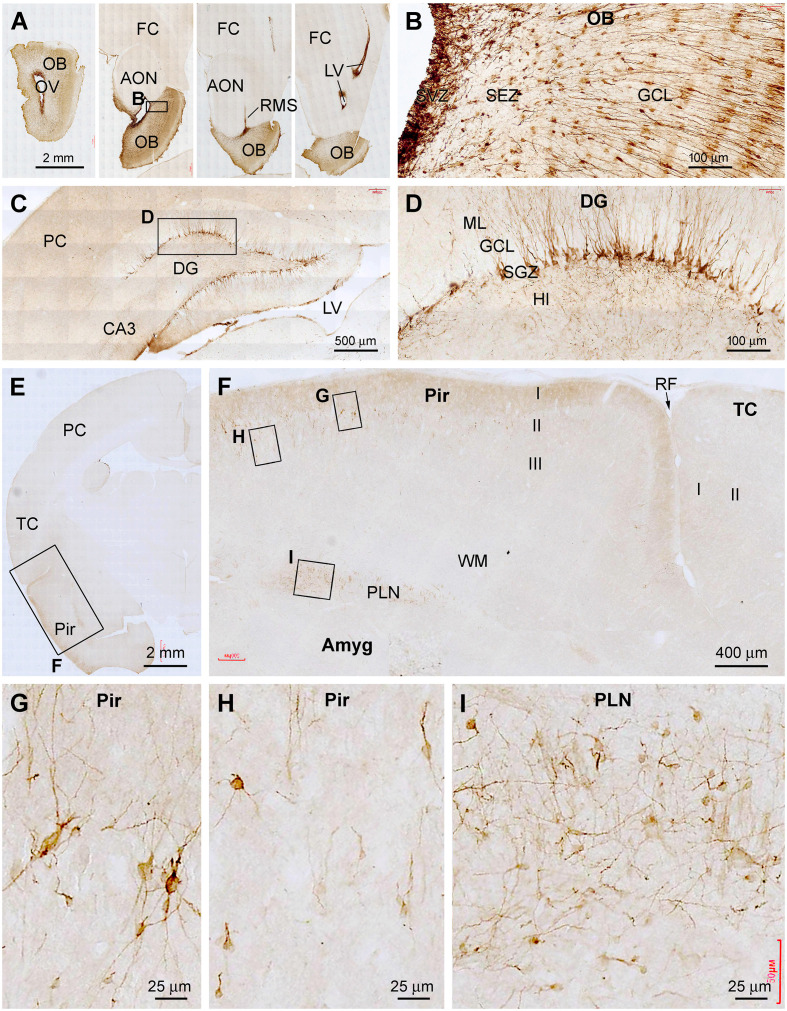
Representative images illustrating DCX+ cellular profiles in the forebrain in a 2 years-old Chinese tree shrew. Panel **(A)** shows the labeling in the olfactory bulb (OB) and rostral migratory stream (RMS) at four coronal levels, noting the open olfactory ventricle (OV) inside the bulb. Panel **(B)** shows the labeled cells densely packed at the subventricular zone (SVZ), with migrating granule cells from the subependymal zone (SEZ) to the granule cell layer (GCL). Panel **(C)** shows labeled cells in the hippocampal dentate gyrus (DG), which are developing granule cells primarily resided along the subgranular zone (SGZ) **(D)**. Panels **(E,F)** show DCX+ immature neurons localized selectively in the piriform cortex (Pir) over layer II, but not in the adjoining temporal neocortex. Panels **(G–I)** show high power views of DCX+ neurons in the piriform cortex and amygdala (Amyg), which are remarkably heterogeneous in somal size, shape, and labeling intensity. Other abbreviations are as defined in Figures 1–3. Scale bars are as indicated.

The topographic distribution pattern of DCX IR in the brains of 1–2 yr-old tree shrews was the same as described above. Representative images obtained from the four above-mentioned forebrain regions are shown in Figure 4. Strongly labeled cells were present in SVZ and SEZ deep to the GCL, and across the GCL throughout the rostrocaudal dimension of the OB (Figure 4A). The labeled somata in the GCL had radially arranged processes, implicating that they were migratory immature granule cells (Figure 4B). In the DG, DCX IR was concentrated along the SGZ (Figures 4C,D), which appeared to be less densely packed as seen in the 2–3 mo-old animals (Figures 2G,H). Across the cerebrum, DCX+ cells were observed in the Pir over layer II, while essentially no labeled profiles were found in the neocortical areas (Figures 4E–H). It also appeared that the overall amount of DCX IR in the Pir was not as great as seen in the 2–3 mo-old animals (Figures 2A,E,F). Furthermore, DCX IR was clearly observed in the Amyg over the area representing the PLN (Figures 4E,F,I). On closer examination (Figures 4G–I), the labeled cells exhibited a large extent of variability in labeling intensity, size and shape, and dendritic arborization. Most of the strongly labeled neurons had an oval or round soma with their longer diameter less than 20 μm (mostly less than 10 μm). The small neurons were bipolar and unipolar in shape. However, DCX+ cells with larger somal sizes were also present, which had multiple dendritic processes and exhibited a multipolar somal shape (Figures 4G–I).

### Distribution of DCX IR in the Brains of 5–6 Years-Old Animals

**Figure 5:**
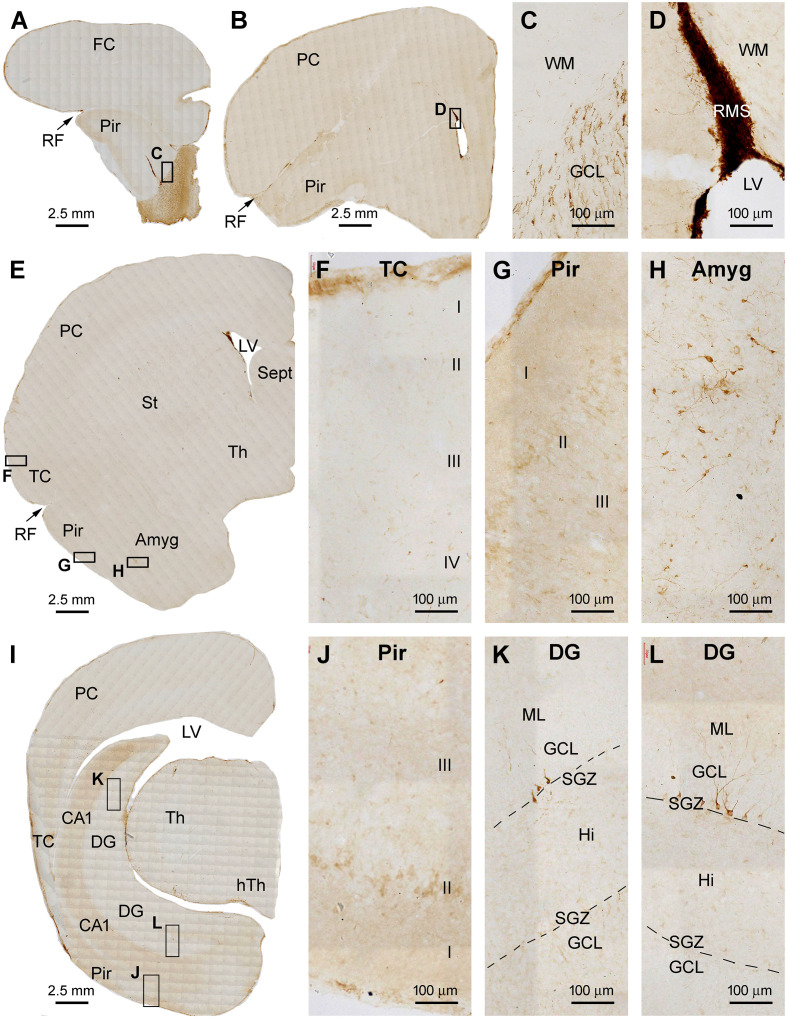
Representative images illustrating DCX+ cellular profiles in the forebrain in a 6 years-old Chinese tree shrew. Overall, the amount of labeling in the forebrain structures is apparently reduced relative to that seen in Figure 4. Panels **(A–D)** are low and high magnification images illustrating the labeling in the olfactory bulb (OB) in the granule cell layer (GCL), and along the rostral migratory stream (RMS). Panels **(E–G)** are low and high magnification images at the level of the septum. There are no labeled cells in the temporal and parietal neocortex **(E,F)**. However, some lightly stained neurons are seen in the piriform cortex in layers II **(E,G)**, while some distinctly labeled neurons remain in the amygdala **(E,H)**. Panel **(I)** is the image of the immunolabeled section passing the temporal hippocampus, with labeled DCX+ cells seen in the piriform cortex **(J)** and a few clusters of labeled granule cells remained in the dentate gyrus **(K,L)**. Abbreviations are as defined in Figures 1–3. Scale bars are as indicated.

A major finding in the brains of 5–6 yr-old tree shrews involved an apparent reduction in the amount of DCX IR across the forebrain regions relative to the above two age groups. Figure 5 shows low and high magnification images of immunolabeled sections from a 6 yr-old animal. In the OB, DCX+ cells were still present in the SEZ and GCL, while the overall amount of IR was noticeably reduced relative to the 1–2 yr-old animals. DCX+ cells at the SVZ were arranged as a thin layer surrounding the LV, while the labeling around the RMS appeared as a thinner band in comparison with that in the animals in the younger age groups (Figures 5A–E). In the Pir, some lightly labeled neuronal somata were seen over layer II. Again, no specific labeling was seen across all the neocortical regions (Figures 5D–G,I,J). It should be noted that the DCX+ neurons in layer II of the Pir were mostly stained lightly to moderately in the 5–6 yr-old animals, with those in small somal size and with heavily labeled dendrites reduced relative to the two other age groups (Figures 5G,J). However, both strongly and weakly labeled neuronal profiles persisted in the PLN, and they showed a large degree of variability in somal size and shape (Figure 5H). In comparison with the 2–3 mo- and 1–2 yr-old groups, DCX+ cells in the DG were apparently reduced in 5–6 yr-old animals. Thus, across the entire hippocampal formation, only a small number of DCX+ cells was seen in the SGZ by examining the sections at high magnifications. These cells were arranged as separate small clusters (Figures 5I,K,L).

### Quantitative Assessment on Age-Related Decline of DCX IR

**Figure 6:**
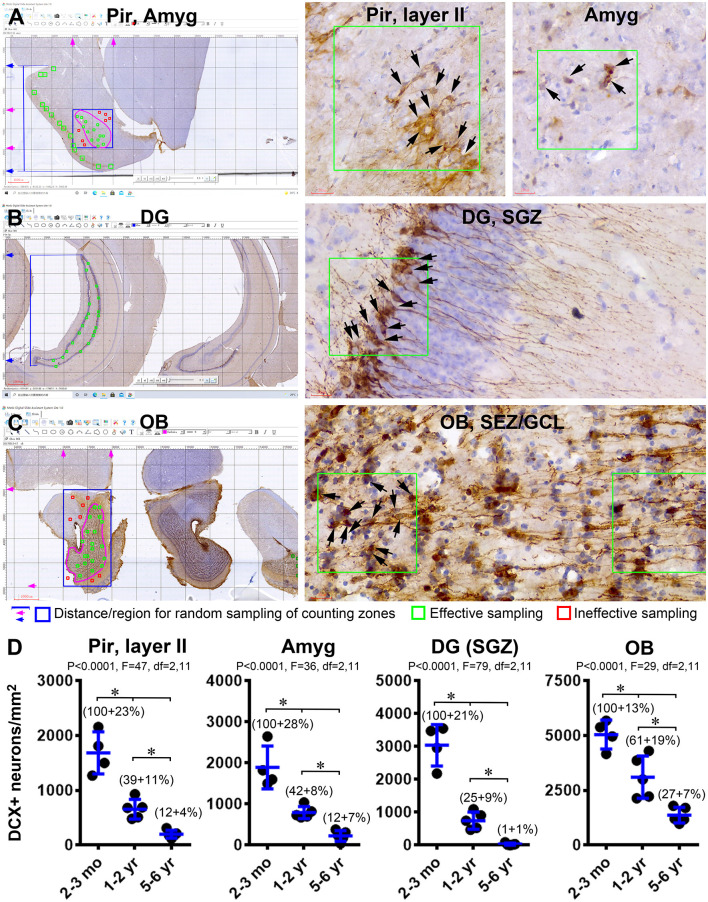
Quantification of age-related decline of DCX+ immature neurons in the forebrain of Chinese tree shrews. Panels **(A–C)** illustrate the methodology for cell count in randomly determined zones in the piriform cortex (Pir) **(A)**, amygdala (Amyg) **(A)**, dentate gyrus (DG) **(B)**, and the olfactory bulb **(C)**. Counting zones (200 × 200 μm^2^) intersecting with layer II in the Pir are randomly generated based on the distance scales from the piriform sulcus to the ventral border of the cerebrum (marked with blue lines and arrows in **A**). Counting zones (100 × 100 μm^2^) in the Amyg are randomly generated in a rectangular area (boxed with blue lines) covering the entire amygdalar complex (pink circle). Only the zones (marked in green) within this tear-drop-shaped nucleus are considered as effective samples, whereas the zones (marked in red) outside its anatomical border are considered as ineffective sampled sites. Similarly, the counting zones (100 × 100 μm^2^) intersecting with the subgranular zone (SGZ) in the DG are randomly generated based on the distance scales from dorsal and ventral edges of the granule cell layer (GCL) **(B)**. The effective sampling zones inside the bulb are across the granule cell layer (GCL) and the subependymal zone (SEZ) (pink circle), which are generated randomly within a rectangular area (marked with blue lines) covering the entire bulb. Panel **(D)** plots the means of DCX+ cell densities in the four regions from individual animals arranged in the three age groups (*n* = 4 in the 2–3 mo-old group, *n* = 5 in 1–2 yr-old group, and *n* = 5 in the 5–6 yr-old group). The normalized means relative to the youngest group are also provided in the graphs. Statistical results and significant intergroup differences (*) based on the one-way ANOVA test with Bonferroni’s pair-wise multiple comparisons are as indicated in individual graphs.

As graphically illustrated in Figure 6, we estimated the extent of age-related decrease in the number or density of DCX+ neurons in the Pir, Amyg, DG, and OB using a modified stereological method by taking advantage of the grid addition option on the Motic image analysis system. The cell densities in the Pir and DG were counted in tagged square zones placed along layer II (200 × 200 μm^2^) and the SGZ (100 × 100 μm^2^) according to the randomly generated locations in reference to the Y-axis of the image, giving that the labeled cells are distributed in a “linear” manner (i.e., along layer II and GCL) (Figures 6A,B). The cell densities in the OB and Amyg were counted in tagged square zones (100 × 100 μm^2^) randomly determined based on both the × and Y axes of the grid system, given the distribution of DCX+ neurons across these regions in a “spreading” manner (Figures 6A,C). It should be noted that, for the Amyg and OB, the random grid selection also gave rise to sampling sites that were outside the anatomical regions applicable for quantification (Figures 6A,C, the squares marked in red, for illustrative explanation), which, accordingly, were excluded from use in the cell counting because they represented “ineffective” sampling. It should be further noted that, because the DCX+ neurons could be fairly crowded in the analyzed forebrain regions especially in the young age group, we carried out the cell count using hematoxylin-counterstained sections in which the co-labeled nucleus could help the differentiation between neighboring DCX+ neurons (right panels of Figures 6A–C).

The mean density (mean ± S.D., same below) of DCX+ neurons in layer II of the Pir was 1,684 ± 385 cells/mm^2^ in the 2–3 mo-old group (*n* = 4), 685 ± 180 cells/mm^2^ in the 1–2 yr-old group (*n* = 5), and 100 ± 70 cells/mm^2^ in 5–6 yr-old group (*n* = 5), respectively. Normalized to the mean of the youngest group, the percentage values were 100 ± 22.8%, 39.1 ± 10.7%, and 11.9 ± 4.2% in the 2–3 mo-old, 1–2 yr-old, and 5–6 yr-old groups, respectively. The means were significantly different among the three groups (*P* < 0.0001; df = 2, 11; *F* = 46.6. One-way ANOVA). Bonferroni’s multiple comparison (*post hoc*) indicated that the means were significantly different between each paired two age groups (Figure 6D). The values in the Amyg were 1,892 ± 520 cells/mm^2^ (100 ± 27.5%), 794 ± 520 cells/mm^2^ (41.9 ± 7.8%) and 228 ± 130 cells/mm^2^ (12.1 ± 6.9%) in the 2–3 mo, 1–2 yr and 5–6 yr groups, respectively. There was an overall significant difference between the means of the three groups (*P* < 0.0001; df = 2, 11; *F* = 35.6), as well as significant differences when the means of a given two age groups were compared individually with *post hoc* test (Figure 6D). Values in the OB were 5,045 ± 660 (100 ± 13.1%), 3,104 ± 660 (61.5 ± 19.1%), and 1,370 ± 357 (27.2 ± 7.1%) cells/mm^2^in the three age groups in the same order as listed above, with the means significantly different in the three groups (*P* < 0.0001; df = 2, 11; *F* = 29.8), and between each paired age groups by *post hoc* test (Figure 6C). The values of DCX+ cells in the SGZ of the DG were 3,040 ± 631 (100 ± 20.8%), 747 ± 261 (24.6 ± 8.6%), and 29 ± 35 (1.0 ± 1.1%) cells/mm^2^ in the three age groups, respectively. An overall difference existed among the age groups (*P* < 0.0001; df = 2, 11; *F* = 79.9), with *post hoc* tests showed a significant age-related decline in the amount of labeling (Figure 6D).

### Double Immunofluorescent Analysis of DCX+ Cells in the Piriform Cortex and Amygdala

**Figure 7:**
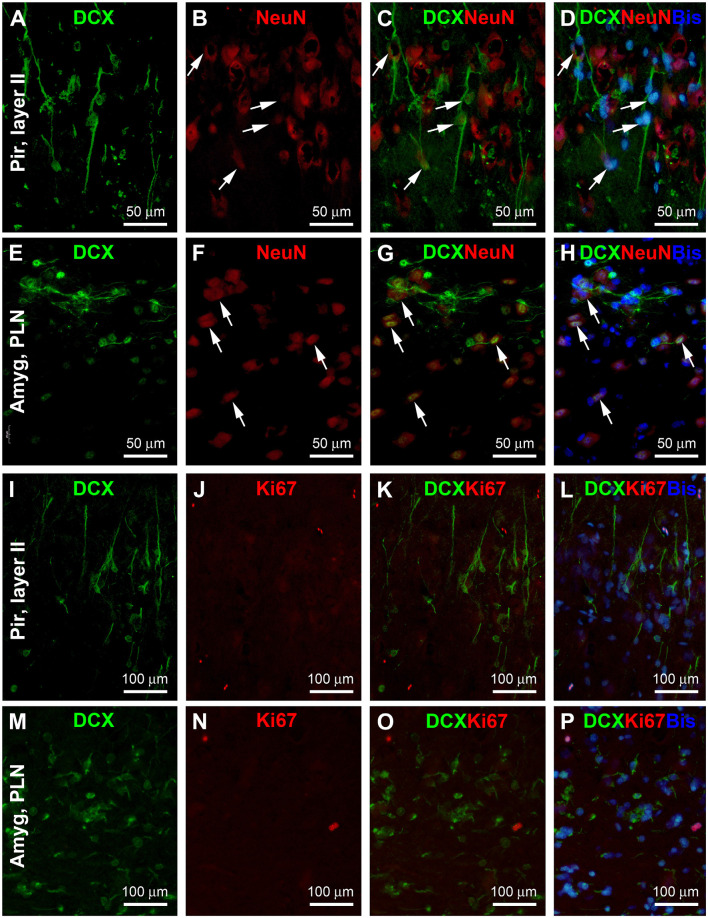
Double immunofluorescent characterization of DCX colocalization with the neuron-specific nuclear antigen (NeuN) and Ki67 in layer II of the piriform cortex (Pir) and paralaminar nucleus (PLN) of the amygdala (Amyg), with bisbenzimide (Bis) used to mark the cell nuclei (assessed in 2 years-old adult tree shrews). In both areas, there exists a partial colocalization of NeuN among the DCX+ cells (**A–H**, examples are pointed by arrows), whereas no Ki67 labeling is seen in the DCX+ cells **(I–P)**. Image locations, fluorescent channels, and scale bars are as indicated in the panels.

Many previous studies have explored the differentiation/maturation and the possibility of the local genesis of layer II and amygdalar DCX+ neurons (e.g., Liu et al., 2008; Xiong et al., 2008, 2010; Luzzati et al., 2009; Cai et al., 2009; Srikandarajah et al., 2009; Patzke et al., 2014; Chawana et al., 2016; Piumatti et al., 2018). In the present study, we carried DCX/NeuN double immunofluorescent experiments using sections from the 1–2 years-old animals (Figure 7). There was a partial colocalization of NeuN among the DCX+ neurons in both the Pir (Figures 7A–D) and Amyg (Figures 7E–H), particularly in the cells with relatively large somal size and light labeling intensity. On the other hand, the small-sized ones with bright DCX immunofluorescence lacked NeuN labeling. No DCX/Ki67 colocalization was observed in the same cells in the Pir (Figures 7I–L) and Amyg (Figures 7M–P). Nonetheless, a few Ki67+ nuclear profiles could be identified in both areas, often arranged in pairs or in clusters.

### Topographic Distribution of DCX IR in Sagittal and Horizontal Cerebral Sections

**Figure 8:**
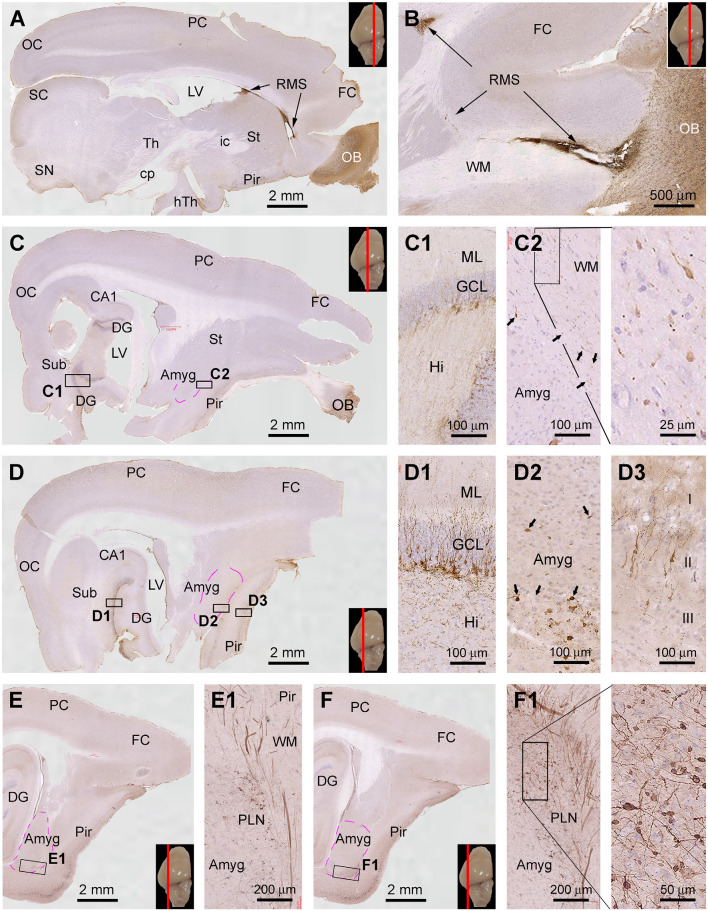
Topographic distribution of DCX+ cellular profiles in sagittal cerebral sections from a 6-month-old animal, with hematoxylin counterstain. The approximate medial to lateral planes of the sections in reference to the top view of the brain are marked with a red line in the image panels. Framed areas in the low magnification images are enlarged as indicated. The area of the amygdalar complex (Amyg) is marked with broken purple lines **(C–F)**. Panels **(A,B)** show the immunolabeling in the subventricular zone (SVZ) forming the rostral migratory stream (RMS) extending into the olfactory bulb (OB). Panels **(C,D)** show the distribution of DCX+ neurons in the hippocampal dentate gyrus (DG), Amyg, and piriform cortex (Pir), with the framed areas enlarged as **(C1,C2)**, and **(D1–D3)** as indicated. Panels **(E,F)** are images at more lateral planes, in which the DCX+ cells in the Amyg are distinctly present in the area of the paralaminar nucleus (PLN) **(E1,F1)**. Additional abbreviations: FC, frontal neocortex; PC, parietal neocortex; OC, occipital neocortex; Th, thalamus; hTh, hypothalamus; St, striatum; Sub, subiculum; GCL, granule cell layer; ML, molecular layer; Hi, hilus; WM, white matter. Scale bars are as indicated.

**Figure 9:**
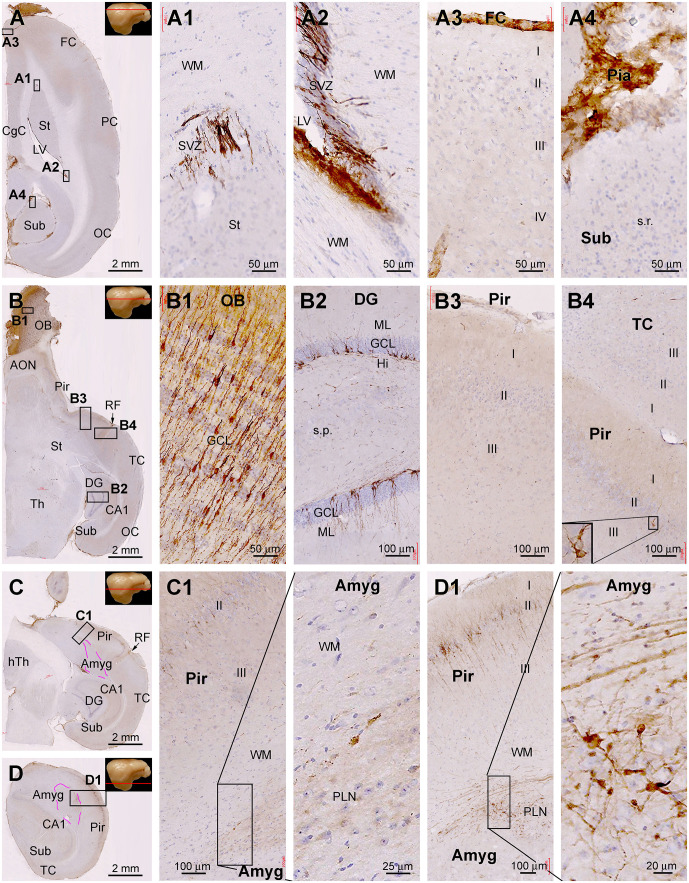
Topographic distribution of DCX+ cellular profiles in hematoxylin-counterstained horizontal cerebral sections from a 4-year-old animal. The approximate levels of the sections relative to the lateral view of the brain are marked with a red line in the image panels. Framed areas in the low magnification panels are enlarged as indicated. The area of the amygdalar complex (Amyg) is marked with broken purple lines **(C,D)**. In the most dorsal level section, DCX labeling is seen at the subventricular zone lining the lateral ventricle surrounded by the stratum (St) and cingulate cortex (CgC) **(A,A1,A2)**. There are no DCX+ cells in the frontal neocortex **(A3)** and subiculum **(A4)**, with no specific labeling likely related to the meninge **(A3,A4)**. In the section at the level of the olfactory bulb **(B)**, DCX+ cells are found across the granule cell layer of the bulb **(B1)**, the subgranular zone of the dentate gyrus **(B2)**. Note that in the piriform cortex (Pir), no DCX+ cells are found in the rostral segment **(B3)**, while a few cells are present in the caudal segment near the rhinal fissure (RF) **(B4)**. At the basal levels of the cerebrum, a large number of DCX+ cells occur in the PLN of the amygdala **(C1)**, while labeled cells are also abundantly present over layer II in the Pir **(D1)**. Additional abbreviations are as defined in Figure 8. Scale bars are as indicated.

During the course of manuscript review, it was advised to also examine the topographic distribution of DCX IR in the forebrain at the sagittal and horizontal planes of the cerebrum. We obtained brains from three animals aged at 6 mo, 1 yr, and 4 yr to accomplish this goal. Hemibrains from each animal were cut into parasagittal and horizontal sections, with DCX immunolabeling data from the 6 mo- and 4 yr-old animals illustrated in Figures 8 and 9.

Thus, in the medial parasagittal sections from the 6 mo-old animal, DCX IR was distinct only in the rostral portion of the forebrain, occurring in the OB and SVZ surrounding the anterior part of the LV (Figures 8A,B). It was possible to trace the IR from the SVZ, along the RMS, and further into the OB (Figure 8B). In middle parasagittal sections, DCX IR in the hippocampal DG was prominent (Figures 8C,D), with the labeled immature granule cells densely packed along the SGZ (Figures 8C1,D1). At these levels, a small amount of DCX+ cellular profiles could be identified at high magnifications in the Amyg near the white matter of the piriform cortex (Figures 8C,C2,D,D2). These labeled cells were generally small in size and had a unipolar or bipolar somal shape (Figures 8C2,D2). Also in these section levels, DCX+ neuronal perikarya were seen in the Pir, which showed intensive IR in the somata and dendritic branches extending into layer I (Figure 8D3). In comparison, the most distinctive DCX IR in the Amyg was seen in parasagittal sections passing the lateral portion of the cerebrum (Figures 8E,E1,F,F1). Thus, a fairly large group of labeled cells, as well as thick chain-like structures, were present around the junction of the basal and posterior borders of the amygdalar complex (Figures 8E1,F1). It should be noted that DCX+ cellular profiles were also mostly densely packed in the Pir at these section levels (Figures 8E,F).

Assessed in horizontal cerebral sections from the 4 yr-old animal (Figure 9), DCX+ cells were present at the SVZ surrounding the dorsal part of the LV approximately around the level of the dorsal surface of the OB (dorsal-level horizontal sections, Figures 9A,A2,A3). There were no DCX+ neuronal profiles in the neocortical areas from the frontal, parietal to occipital lobes (Figures 9A,A1). Some non-specific labeling was seen around the pia or meninge in the sections (Figures 9A,A1,A4). At the middle-level horizontal sections (passing the OB), DCX IR was distinctly seen across the OB layers as well as along the SGZ of the DG (Figures 9B,B1,B2). There were a few DCX+ cells in layer II of the Pir, which were preferentially located in the part close to the RF (i.e., the part bordering with the temporal neocortex) (Figures 9B,B3,B4). In the ventral or basal level of horizontal sections (approximately below the ventral surface of the OB) (Figures 9C,D), DCX IR was seen much more clearly in the Amyg and the Pir, with the cells and chain-like structures in the Amyg also occurred most densely next to the Pir (Figures 9C1,D,D1).

## Discussion

### Topographic Feature and Age-Related Decrease of DCX+ Cells in Tree Shrew Forebrain

As denoted in the “Introduction” section, DCX+ neurons, or alike, in the cerebral cortex have been reported in many mammals, while there are noticeable species differences in regard to their topographic distribution. Specifically, DCX+ immature neurons are essentially restricted to the Pir in small laboratory rodents, whereas they are present in the neocortex as well in many middle- to large-brained mammals (Bonfanti et al., 1992; Seki and Arai, 1993; Fox et al., 2000; Nacher et al., 2001; Xiong et al., 2008; Cai et al., 2009; Luzzati et al., 2009; Srikandarajah et al., 2009; Zhang et al., 2009; Chawana et al., 2013, 2016; Patzke et al., 2014; Liu et al., 2018; Piumatti et al., 2018; La Rosa et al., 2020a, b). Notably, in the hedgehogs, DCX+ cells appear to be restricted to layer II within the piriform cortex (Alpár et al., 2010; Bartkowska et al., 2010). Based on the assessment of DCX IR in animals at life stages ranging approximately from early childhood, youth to mid-age adulthood relative to humans, we show here that the overall topographic pattern of these cortical immature neurons in Chinese tree shrew is similar to that seen in small rodents, and likely the insectivore hedgehogs as well. It should be noted that, based on collective assessment in frontal, sagittal, and horizontal cerebral sections, the distribution of layer II DCX+ cells within the piriform cortex of Chinese tree shrew is actually topographically differential. Thus, these immature neurons are mostly localized to the region surrounding the amygdalar complex, but become rare or are absent as moving to more rostral, caudal as well as dorsal and ventral parts of the paleocortex.

As also aforementioned, DCX+ immature neurons, or alike, are prominently present in the amygdala in primates and are also found in various nonprimate species (Francis et al., 1999; Yachnis et al., 2000; Fudge, 2004; Angata et al., 2007; Cai et al., 2009; Zhang et al., 2009; Alpár et al., 2010; Fudge et al., 2012; Martí-Mengual et al., 2013; Patzke et al., 2014; Chawana et al., 2016; Fasemore et al., 2018; Limacher-Burrell et al., 2018; Liu et al., 2018; Piumatti et al., 2018; Sorrells et al., 2019; Bernier et al., 2020). Comparatively, DCX+ cells are less prominently present in the amygdala in mice, rats, guinea pigs, and rabbits (Nacher et al., 2001; Xiong et al., 2008; Luzzati et al., 2009; Jhaveri et al., 2018). We report here that DCX+ neurons occur in the amygdala in Chinese tree shrews, most densely distributed around its border to the white matter of the piriform cortex, which is consistent with the anatomical location of the PLN in this species (Flügge et al., 1994). It should be noted that we could not identify an apparent cellular stream from the SVZ surrounding the temporal LV, whereas the RMS from the SVZ around the anterior LV to the OB could be clearly observed in frontal and sagittal cerebral sections.

Aged-related decrease in the number or density of DCX+ cells in the SVZ, RMS/OB, and SGZ has been shown in various species (Seki and Arai, 1995; Kuhn et al., 1996; Gould et al., 1999; Leuner et al., 2007; Knoth et al., 2010; Lugert et al., 2010; Encinas et al., 2011; Mobley et al., 2013; Capilla-Gonzalez et al., 2015; Dennis et al., 2016; Smith and Semënov, 2018; Sorrells et al., 2018; Moreno-Jiménez et al., 2019, 2021; Tobin et al., 2019; Sorrells et al., 2021). Likewise, DCX+ neurons are reportedly reduced in the piriform cortex, neocortex, and amygdala with age in several mammals (Xiong et al., 2008; Cai et al., 2009; Zhang et al., 2009; Piumatti et al., 2018). In the present study, we carried out randomized cell count on the DCX+ neurons in the piriform cortex and amygdala, as well as the olfactory bulb and dentate gyrus with adult neurogenesis. The overall densities of DCX+ neurons in the four analyzed regions were reduced in a step-wise manner in the three age groups. The microscopic observation and cell density quantification indicate that DCX+ neurons persist in the piriform cortex and amygdala in the tree shrews into older ages relative to their counterpart in the hippocampal dentate gyrus. Thus, the occurrence of DCX+ neurons in the piriform cortex and amygdala in old shrews appear similar to the cases observed in nonhuman primates (Zhang et al., 2009), and lately in humans as well (Sorrells et al., 2021).

### Potential Phylogenic Implication for the Distribution of DCX+ Immature Neurons in the Tree Shrew Cerebral Cortex and Amygdala

The expression of DCX and other molecular markers typically associated with neuronal differentiation in morphologically heterogeneous cortical and amygdalar neurons in the adult and even aged mammalian brains not only is phenomenal but also could implicate a certain fundamental neurobiological principle yet to be elucidated. DCX+ cells in tree shrew piriform cortex and amygdala appear to be a group of immature neurons based on their partial colocalization with NeuN, as suggested previously (see review La Rosa et al., 2020b). During mammalian evolution, both the neocortex and amygdala undergo progressive expansion, which may support more advanced cognitive, emotional, and social interaction activities. Thus, the neocortex and amygdala are more complex neuroanatomically, developmentally, and cellularly in primates than in low mammals, especially the most commonly studied laboratory mice and rats. The PLN is considered the newest amygdalar subdivision mostly expanded in primates (Kordower et al., 1992; Ulfig et al., 2003; deCampo and Fudge, 2012; Fudge et al., 2012).

Relating to neocortical and amygdalar evolution, it is of interest that three shrews show a rodent-like pattern in the distribution of DCX+ immature cortical neurons but a primate-like pattern involving the localization of the immature neurons in the amygdala, while guinea pigs exhibit an opposite scenario (Xiong et al., 2008). As denoted earlier, the Chinese tree shrew possesses a phylogenetic profile closer to that of primates than rodents (Fan et al., 2013, 2019). Notably, although being a rodent in traditional morphological taxonomy, guinea pigs also appear to be fairly different from mice and rats in phylogenetic perspectives (Graur et al., 1991; D’Erchia et al., 1996; Sharman et al., 2013).

Based on existing reports involving the presence and topographic distribution of DCX+ immature neurons in the forebrain among mammals, as elaborated in the “Introduction” section, tree shrews and guinea pigs appear to have both developed some intermediate characteristics in regard to cerebral cellular constitution between small rodents and primates, as evidenced by the addition of DCX+ immature neurons in the neocortex and amygdala, which appears to occur in an “imbalance” manner between two species. Given that guinea pigs and tree shrews exhibit somewhat “mixed” small rodent-like and primate-like topographic distribution patterns of DCX+ cortical and amygdalar neurons, these two animals could be useful to address certain neuroanatomical and functional issues involving the cerebral immature neurons in relevance to brain evolution, development and plasticity.

## Conclusion

The present study has characterized DCX+ neurons in several forebrain regions in Chinese tree shrews. DCX+ neurons are present in the established neurogenic sites, namely the SVZ, SEZ, RMS, OB, and SGZ. In the cerebral cortex, DCX+ neurons are restricted to layer II in the piriform cortex, which is comparable to the distribution pattern of these neurons in mice and rats. A significant population of DCX+ neurons is present in the amygdala, which resembles the distribution pattern of these neurons in primates. We also show that the age-related decline in the amount of DCX+ neurons proceeds more significantly in the hippocampal dentate gyrus relative to the olfactory bulb, piriform cortex, and amygdala in the tree shrews.

## Data Availability Statement

The original contributions presented in the study are included in the article/Supplementary Material, further inquiries can be directed to the corresponding author/s.

## Ethics Statement

The animal study was reviewed and approved by the Institutional Animal Care and Use Committee of Central South University Xiangya School of Medicine.

## Author Contributions

J-QA, CY, and TT carried out immunohistochemical preparations. RL, JJ, CY, and RB contributed to histological preparation. BZ contributed to cell quantification. J-QA, TT, and X-XY contributed to data analysis. ET, Y-GY, and X-XY provided funding. TT and X-XY designed the study and wrote the manuscript. All authors contributed to the article and approved the submitted version.

## Conflict of Interest

The authors declare that the research was conducted in the absence of any commercial or financial relationships that could be construed as a potential conflict of interest.

## Publisher’s Note

All claims expressed in this article are solely those of the authors and do not necessarily represent those of their affiliated organizations, or those of the publisher, the editors and the reviewers. Any product that may be evaluated in this article, or claim that may be made by its manufacturer, is not guaranteed or endorsed by the publisher.
