# Supplementary figures and images for: Doublecortin-Expressing Neurons in Chinese Tree Shrew Forebrain Exhibit Mixed Rodent and Primate-Like Topographic Characteristics
Source: Front Neuroanat. 2021 Sep 16;15:727883. doi: 10.3389/fnana.2021.727883 (PMC8481370; doi:10.3389/fnana.2021.727883)

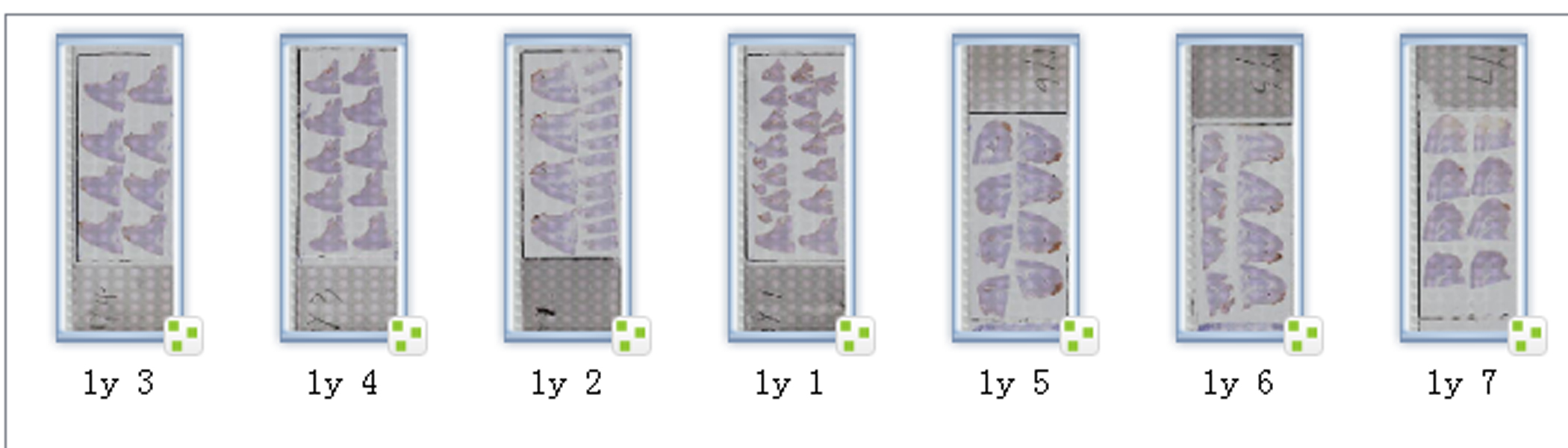

Supplement: Supplementary file 1 [file Data_Sheet_1.ZIP › 1 year-old sagittal images.jpg]

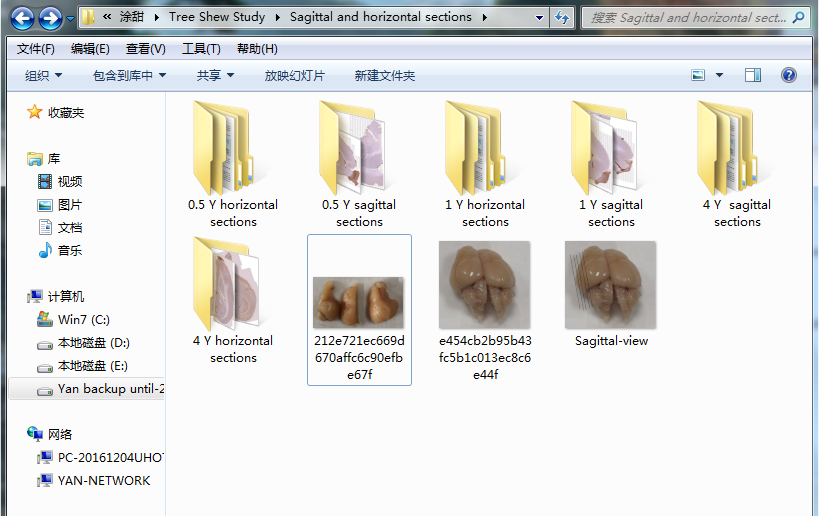

Supplement: Supplementary file 1 [file Data_Sheet_1.ZIP › New data files-.jpg]

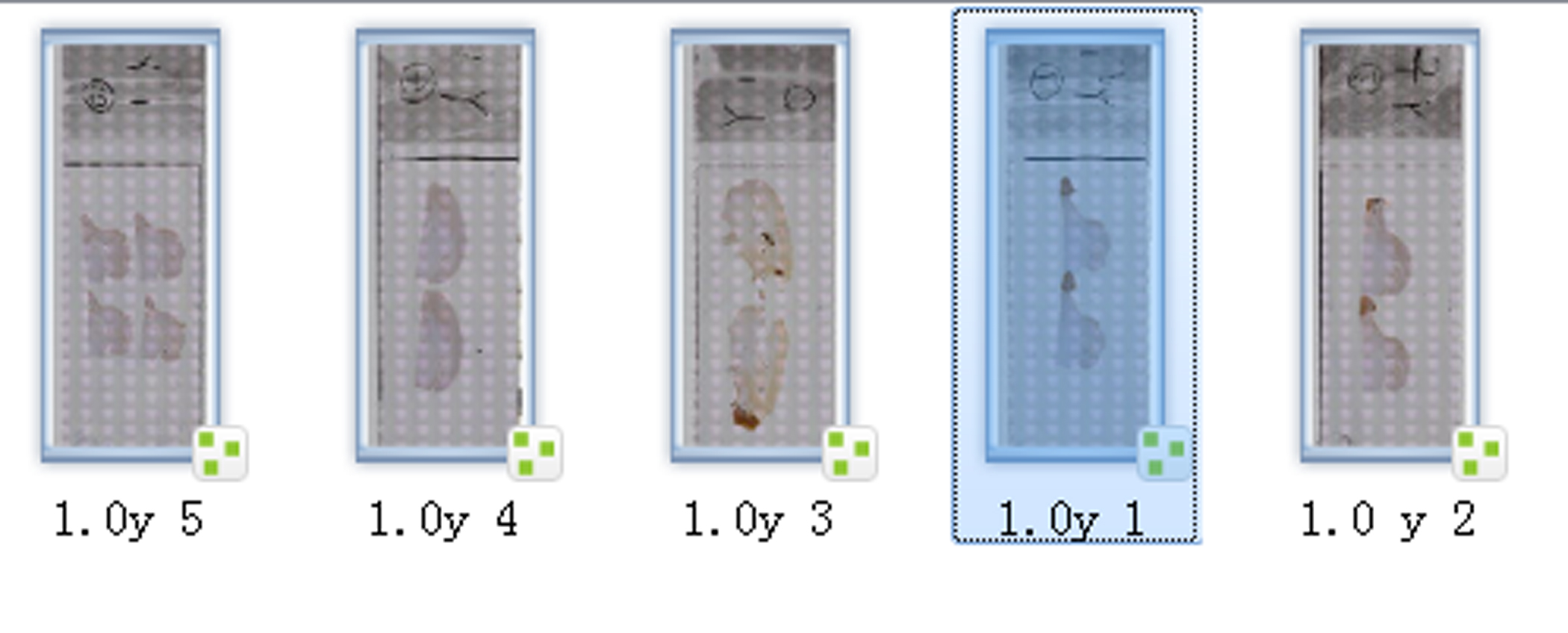

Supplement: Supplementary file 1 [file Data_Sheet_1.ZIP › 1 year-old Horizontal images.jpg]
